# The cyclin‐dependent kinase G group defines a thermo‐sensitive alternative splicing circuit modulating the expression of Arabidopsis *ATU2AF65A*

**DOI:** 10.1111/tpj.13914

**Published:** 2018-05-10

**Authors:** Nicola Cavallari, Candida Nibau, Armin Fuchs, Despoina Dadarou, Andrea Barta, John H. Doonan

**Affiliations:** ^1^ Max F. Perutz Laboratories Medical University of Vienna Vienna Biocenter, Dr Bohr‐Gasse 9/3 A‐1030 Wien Austria; ^2^ Institute of Biological, Environmental and Rural Sciences Aberystwyth University Aberystwyth SY23 3EB UK; ^3^ Present address: Institute of Science and Technology Austria Am Campus 1 3400 Klosterneuburg Austria

**Keywords:** alternative splicing, thermo‐sensitivity, cyclin‐dependent kinases, Arabidopsis

## Abstract

The ability to adapt growth and development to temperature variations is crucial to generate plant varieties resilient to predicted temperature changes. However, the mechanisms underlying plant response to progressive increases in temperature have just started to be elucidated. Here, we report that the cyclin‐dependent kinase G1 (CDKG1) is a central element in a thermo‐sensitive mRNA splicing cascade that transduces changes in ambient temperature into differential expression of the fundamental spliceosome component, *ATU2AF65A*. *CDKG1* is alternatively spliced in a temperature‐dependent manner. We found that this process is partly dependent on both the cyclin‐dependent kinase G2 (CDKG2) and the interacting co‐factor CYCLIN L1 (CYCL1), resulting in two distinct messenger RNAs. The relative abundance of both *CDKG1* transcripts correlates with ambient temperature and possibly with different expression levels of the associated protein isoforms. Both CDKG1 alternative transcripts are necessary to fully complement the expression of *ATU2AF65A* across the temperature range. Our data support a previously unidentified temperature‐dependent mechanism based on the alternative splicing (AS) of *CDKG1* and regulated by CDKG2 and CYCL1. We propose that changes in ambient temperature affect the relative abundance of *CDKG1* transcripts, and this in turn translates into differential CDKG1 protein expression coordinating the AS of *ATU2AF65A*.

## Significance Statement

One of the most important challenges of modern agriculture is the production of plant varieties that are better adapted to the predicted climatic changes. However, a deep understanding of the ambient temperature response in plants remains incomplete. Here we describe a gene circuit in the cyclin‐dependent kinase family of Arabidopsis able to modulate the activity of downstream targets by adjusting mRNA processing to ambient temperature.

## Introduction

Plants adapt quickly to changes in ambient temperature, optimizing diverse physiological and developmental processes. Understanding the mechanisms underlying this adaptation becomes ever more critical for agriculture in times of climate change (Solomon, 2007; Porter *et al*., 2014). Crops are a vital source of food supply, and small changes in either the average temperature or extreme heat events could have significant impacts on yields (Lobell *et al*., 2008). While heat waves have dramatic effects on productivity of many plant species including cereals (Barnabás *et al*., 2008; Schlenker and Roberts, 2009; Bita and Gerats, 2013; Hatfield and Prueger, 2015), adaptation to moderate fluctuations in temperature, thermo‐morphogenesis, still presents many open questions (Quint *et al*., 2016). Identification of the molecular components involved in temperature modulation of plant development is a primary aim in breeding thermo‐tolerant crop varieties.

Although the coordination of gene expression in response to fluctuating temperatures seems to be conserved across eukaryotes (Kumar and Wigge, 2010), it is less clear whether the sensing mechanisms are also conserved. In mammals, external temperature sensing is mediated by well‐defined thermo‐sensors (Vriens *et al*., 2014), but no similar system has been reported for plants. Strikingly, phytochromes have recently been implicated as thermo‐sensors, specifically regulating plant morphogenesis in response to temperature variations (Jung *et al*., 2016; Legris *et al*., 2016).

Several processes in plants are strongly influenced by temperature at the molecular level, including flowering time and the circadian clock mechanism (Troncoso‐Ponce and Mas, 2012; Gu *et al*., 2013; Song *et al*., 2013; Marshall *et al*., 2016). Moreover, plants are able to integrate environmental perturbations directly by transcription of stress‐responsive genes (Staiger and Brown, 2013; Samanta and Thakur, 2015) and, recently, regulation of pre‐mRNA splicing *per se* was proposed to be part of a ‘molecular thermometer’ adjusting the transcriptome to specific conditions (Capovilla *et al*., 2015). Several genes undergo temperature‐sensitive mRNA maturation (Posé *et al*., 2013; Streitner *et al*., 2013; Airoldi *et al*., 2015; Sureshkumar *et al*., 2016), including splicing regulators (Reddy and Shad Ali, 2011; Verhage *et al*., 2017), and may represent a general mechanism by which temperature modulates gene expression.

Splicing is the removal of intronic (mostly non‐coding) sequences from the primary transcript to form the mature messenger RNA (mRNA). This takes place within a multi‐megadalton complex, the spliceosome, that assembles on the pre‐mRNA and contains five small nuclear ribonucleoprotein particles (snRNPs U1, U2, U4, U5 and U6) and approximately 100 non‐snRNP proteins, including SR proteins and hnRNPs (Krämer, 1996; Graveley, 2000; Zhou *et al*., 2002; Nogués *et al*., 2003; Jurica *et al*., 2004). Multiple transcripts may be produced from a single pre‐mRNA through the combinatorial usage of distinct splice sites in a process called alternative splicing (AS). In mammals and plants, AS affects more than 90% and 63% of intron‐containing genes, respectively (Mollet *et al*., 2010; Zhang *et al*., 2015). Phosphorylation and dephosphorylation of various spliceosome components play a central role in regulating formation, activation and inactivation of the spliceosome and in the control over constitutive and AS (Gui *et al*., 1994; Xiao and Manley, 1997; Misteli *et al*., 1998; Wang *et al*., 1998; Graveley, 2000; Shin and Manley, 2004; Shepard and Hertel, 2009; Kralovicova *et al*., 2011).

Cyclin‐dependent kinases (CDKs) are an evolutionarily conserved family of serine/threonine kinases in Eukaryotes. Initially, CDKs were characterized for their role in cell cycle (Strausfeld *et al*., 1996; Rane *et al*., 1999; Sherr and Roberts, 1999; Kozar *et al*., 2004), but they are also involved in other regulatory pathways like DNA replication and repair (Caspari and Hilditch, 2015; Chadha *et al*., 2016). Moreover, CDKs have been implicated in pre‐mRNA processing through the interaction with several spliceosome components (Ko *et al*., 2001; Hu *et al*., 2003; Loyer *et al*., 2005; Even *et al*., 2006; Cheng *et al*., 2012). In particular, human CDK11 was shown to affect both pre‐mRNA splicing and apoptosis, while retaining a primary role in cell cycle (Loyer *et al*., 2005).

In plants, the CDKG group is the most closely related to human CDK11 (Menges *et al*., 2005; Umeda, 2005). CDKGs are also phylogenetically linked to the enigmatic Ph1 kinase cluster that participates in chromosome pairing in wheat (Doonan and Kitsios, 2009). In Arabidopsis, CDKG proteins are encoded by two closely related genes, *CDKG1* and *CDKG2*. CDKG1 has a role in splicing of a pollen cell wall gene (Huang *et al*., 2013), and in chromosome pairing and recombination at high ambient temperature (Zheng *et al*., 2014). CDKG2 was reported as a negative regulator of flowering and abiotic stress response (Ma *et al*., 2015), but not previously implicated in mRNA regulation.

Given the previously described roles of CDKG1 in splicing (Huang *et al*., 2013) and meiosis (Zheng *et al*., 2014), both inherently temperature‐sensitive processes, we asked whether CDKG1 plays a role in mRNA thermo‐response. Here, we show that CDKG1 is the central element in a thermo‐sensitive cascade that transduces changes in ambient temperature into differential transcript abundance of the target gene *ATU2AF65A*. This mechanism requires the AS of *CDKG1* itself in order to maintain the proper expression pattern of *ATU2AF65A* across the temperature range. Finally, CDKG2 and the associated CYCL1 activate the circuit by adjusting *CDKG1* AS to ambient temperature.

We propose that CDKG1, together with CDKG2 and CYCL1, is part of a molecular thermometer fine‐tuning environmental information into AS of target genes.

## Results

### CDKG1 is required for *ATU2AF65A* AS at ambient temperature

**Figure 1:**
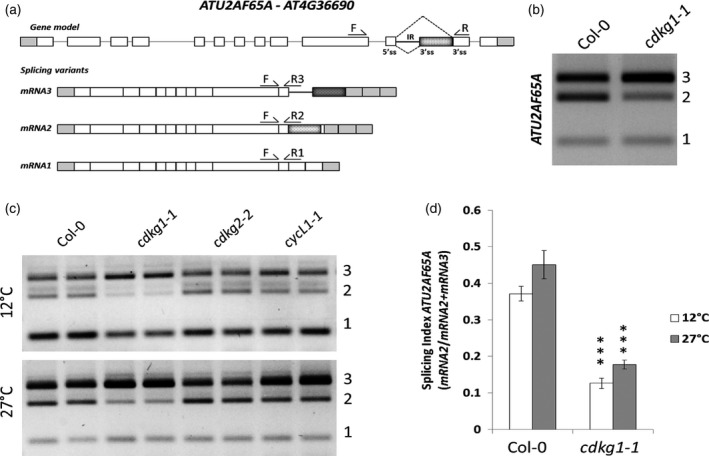
*ATU2AF65A* splicing pattern is affected by temperature and CDKG1. (a) Schematic representation of *ATU2AF65A* locus and mRNA variants, including exons (boxes) and introns (lines). White boxes correspond to coding exons, grey boxes correspond to non‐coding exon sequences (UTRs), dotted boxes correspond to alternative exons, coding or non‐coding in the specific mRNA. Dotted lines represent alternative splicing (AS) events; IR intron retention. Position of primers used for reverse transcriptase‐polymerase chain reaction (RT‐PCR) or RT‐quantitative (q)PCR is indicated (arrows). mRNA1, fully spliced *ATU2AF65A*; mRNA2, partial intron retention; mRNA3, total intron retention. (b) Gel separation of RT‐PCR products using primers F+R as indicated in (a), of *ATU2AF65A* splicing variants in 2‐week‐old Col‐0 and *cdkg1‐1* seedlings, showing defective AS in the mutant line. (c) *ATU2AF65A* splicing pattern after gel separation of RT‐PCR product using primers F+R as indicated in (a) in different mutant backgrounds showing the sensitivity of mRNA variants to temperature shifts. (d) Relative levels of *ATU2AF65A* mRNA2 and mRNA3 estimated by RT‐qPCR. Splicing index (SI) calculates the ratio mRNA2/[mRNA2 + mRNA3] (partial and total intron 11 retention) in Col‐0 and *cdkg1*‐*1* seedlings grown at 12°C (White) or at 27°C (Grey). Each mRNA isoform was detected with the primer as pairs indicated in (a). Data represent means ± SD (*n* ≥ 3). Student's *t*‐test comparing Col‐0 with *cdkg1‐1*: ***P < 0.001.

Several Arabidopsis CDKs have been previously shown to be involved in pre‐mRNA processing (Kitsios *et al*., 2008; Hajheidari *et al*., 2013; Huang *et al*., 2013). On the other hand, splicing factor transcripts are themselves subjected to temperature‐dependent AS (Reddy and Shad Ali, 2011; Verhage *et al*., 2017). Using a panel of genes representing more than 80 potential alternative transcripts, we performed a reverse transcriptase‐polymerase chain reaction (RT‐PCR) screen for splicing events dependent on functional CDKG1 (Table S1). We found that AS of *ATU2AF65A* (*U2 Auxiliary Factor 65A*; Figure 1a for scheme), a constitutive spliceosomal component involved in flowering time regulation (Verhage *et al*., 2017), was affected in the null allele *cdkg1‐1* (Figure 1b). *ATU2AF65A* has a complex splicing pattern of intron 11: intron splicing (mRNA1) and partial (mRNA2) or complete intron retention (mRNA3). Specifically, lack of CDKG1 decreased splicing efficiency with a consequent increase in intron 11 retention in the mature mRNA (mRNA3; Figure 1b).

Because CDKG1 was previously known to have a temperature‐dependent function in meiosis and to be involved in mRNA maturation (Huang *et al*., 2013; Zheng *et al*., 2014), we asked whether its function was required along the environmental temperature range, using *ATU2AF65A* as a gene model. Wild‐type Col‐0 and *cdkg1‐1* mutant seedlings were grown for 2 weeks at 22°C, and then transferred to 12°C or 27°C for 48 h to test the effect of temperature on target pre‐mRNA processing. We included mutant lines for *CDKG2* and *CYCL1* (*cdkg2‐1*;* cycL1‐1*; Zheng *et al*., 2014; Ma *et al*., 2015) to reveal possible compensatory functions or cross‐regulations: *CDKG2* is the only paralogue of *CDKG1* and *CYCL1* is the cognate cyclin in Arabidopsis for both kinases (Van Leene *et al*., 2010).

In wild‐type plants, splicing of *ATU2AF65A* was affected by temperature (Figure 1c), as previously reported (Verhage *et al*., 2017). At 12°C, the fully spliced form (mRNA1) was the most abundant, while at 27°C the complete intron retention form (mRNA3) was dominant. In *cdkg1‐1* plants, the intron 11 splicing defect was more prominent at high temperatures (Figure 1c, lower panel). Moreover, c*ycL1‐1* showed a similar splicing pattern to *cdkg1‐1* with increased intron retention. Interestingly, we did not observe a splicing defect in *cdkg2‐2* plants (Figure 1c). To validate these results, we analysed the levels of each isoform by quantitative RT‐PCR (RT‐qPCR). We confirmed that the three *ATU2AF65A* mRNA variants were differentially regulated by temperature: in wt plants mRNA1 was the most abundant at 12°C, while at 27°C mRNA2 and mRNA3 levels were increased (Figure S1a). Superimposed on the temperature regulation, *cdkg1‐1* showed impaired levels of partial and total intron retention (mRNA2 and mRNA3, decreased and increased, respectively), while the expression of fully spliced mRNA1 was not different from control plants. In the *cycL1‐1* mutant, we also observed increased mRNA3 and mRNA2 levels at both temperatures but these were not compensated by equal mRNA1 reduction, suggesting that transcriptional regulation is also involved (Figure S1a).

To quantify the differences between the mutant *cdkg1‐1* and the wt, we calculated the splicing index (SI) as the ratio between the affected transcripts (mRNA2/[mRNA2 + mRNA3]). In this way, we estimated that the efficiency in splicing of *ATU2AF65A* mRNA was approximately threefold lower in the *cdkg1‐1* mutant than in Col‐0 at both 12°C and 27°C (Figure 1d).

These results indicate that CDKG1 is an important regulator of *ATU2AF65A* mRNA splicing, but there is an additional and partially independent effect of ambient temperature.

### CDKG2 and CYCL1 adjust *CDKG1* AS to ambient temperature

**Figure 2:**
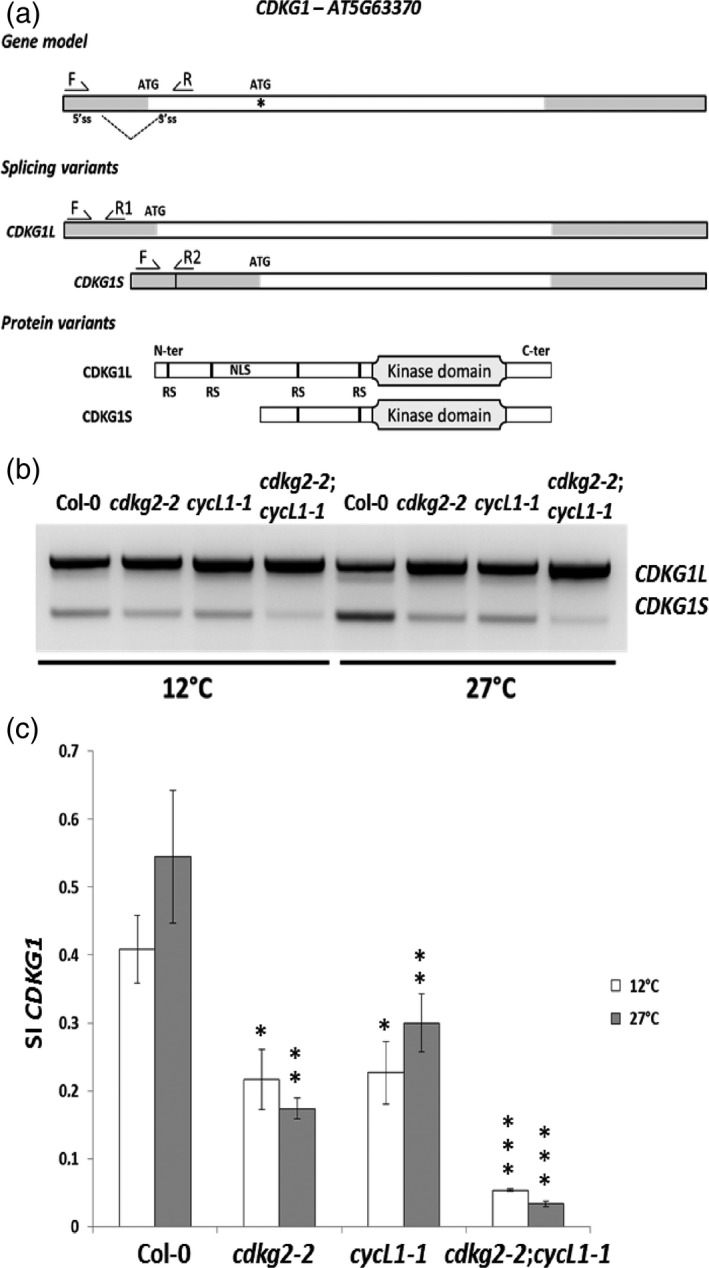
*CDKG1* alternative splicing (AS) is temperature dependent, and requires CDKG2 and CYCL1. (a) Schematic representation of *CDKG1* locus, investigated mRNA and predicted protein variants. White and grey boxes in gene and mRNAs correspond to coding and non‐coding sequences (UTRs), respectively. An alternative start codon (ATG) is indicated in the gene model (asterisk). Dotted lines represent alternative intron 1 splicing event harboring long (L) and short (S) mRNA variants. Position of primers used for reverse transcriptase‐polymerase chain reaction (RT‐PCR) or RT‐quantitative (q)PCR is indicated (arrows). In the protein models, the annotated domains are indicated (RS = arginine/serine‐rich domain for protein–protein interaction; NLS = nuclear localization signal). (b) RT‐PCR products using primers F and R as indicated in (a) after gel separation showing *CDKG1* splicing pattern in Col‐0 and mutant backgrounds at 12°C or at 27°C. Defective AS was evident in the single and double mutant lines for *CDKG2* and *CYCL1*, in seedlings exposed to high temperature. (c) RT‐qPCR analysis of the splicing defect in *CDKG1* measured as the relative ratio *CDKG1S*/[*CDKG1S* + *CDKGL*] (splicing index, SI). Primer position is reported in (a) and each couple is specifically located on the respective mRNA isoform. Data represent means ± SD (*n* = 3). Student's *t*‐test comparing Col‐0 with c*dkg2‐2*,* cycL1‐1* or *cdkg2‐2*;*cycL1‐1* at the respective temperature: ***P < 0.001; **P < 0.01; *P < 0.05.

To explore this further, we next asked whether *CDKG1* mRNA levels themselves fluctuate along the temperature range in order to maintain the proper ratio between *ATU2AF65A* isoforms. Contrary to our expectations, *CDKG1* was constitutively expressed at similar levels both at high and low temperatures in the wild‐type, as well as in the single *cdkg2‐2* and *cycL1‐1* and in the double *cdkg2‐2;cycL1‐1* mutant lines (Figure S2a). However, we noted the presence of several bands for the *CDKG1* transcript in RT‐PCR, suggesting alternative mRNA splicing. While *CDKG1* does not need to undergo splicing to produce a mature coding mRNA, several introns have been annotated in what was considered an intron‐less gene (Figure 2a and Figure S2b; Table S2). Moreover, *CDKG1* alternative introns were preferentially spliced in Col‐0 at higher temperatures, while largely retained at lower ones (Figure 2b and Figure S2b; Verhage *et al*., 2017).

Strikingly, *CDKG1* intron 1 removal was severely impaired in *cdkg2‐2*,* cycL1‐1* and further decreased in the double mutant (Figure 2b). We named the alternative mRNA species as *CDKG1L* (Long) and *CDKG1S* (Short), depending on whether the intron was retained or spliced, respectively. We speculated that this could be a mechanism to fine‐tune *CDKG1* transcript/protein abundance in a temperature‐dependent manner, under the control of CDKG2 and CYCL1.

To quantify this effect, the SI was calculated as the ratio of *CDKG1S*/[*CDKG1S* + *CDKGL*]. In Col‐0, the SI at 27°C was approximately 1.5‐fold that calculated for 12°C, while the SI was decreased in *cdkg2‐2*,* cycL1‐1* (3‐ and 1.8‐fold, respectively) and in the double mutant lines (16‐fold; Figure 2c) where the temperature dependence of the SI was almost completely abolished. No appreciable differences in the annotated AS events for *CDKG2* or *CYCL1* transcripts occurred either at different temperatures or in different backgrounds (Figure S3a), suggesting that temperature regulation was specific for *CDKG1* mRNA.

Removal of *CDKG1* intron 1, located across the 5′UTR and adjacent coding region, erases the primary start codon (ATG). This shorter transcript could use a downstream in frame start codon to produce an uncharacterized CDKG1 protein lacking the nuclear localization signal (NLS) and two (out of four) of the reported Arg/Ser‐rich (RS) interaction domains (Huang *et al*., 2013), based on database annotations (TAIR 10; Figure 2a).

**Figure 3:**
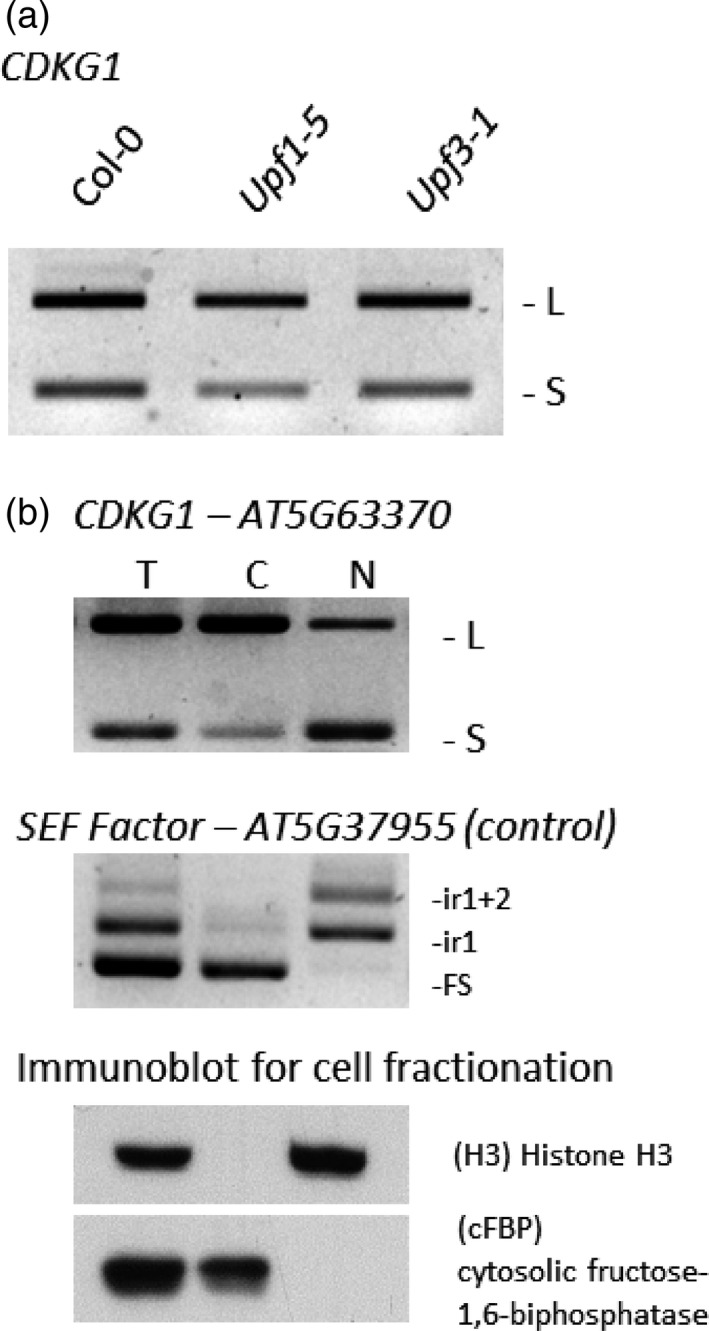
*CDLK1L* and *S* are exported to the cytoplasm and not degraded by the nonsense mediated mRNA decay (NMD) pathway. (a) Reverse transcriptase‐polymerase chain reaction (RT‐PCR) products of *CDKG1* long and short mRNA isoforms in wt and in the NMD defective *upf1‐5* and *upf1‐3* mutant backgrounds. Analysis of the splicing pattern showed no accumulation for any of the transcripts, suggesting no sensitivity to degradation by the NMD pathway. (b) Upper panel, gel separation of RT‐PCR products of *CDKG1* splice variants (*L* and *S*) in fractionated cell extracts. Middle panel, gel separation of RT‐PCR products of the *SEF* factor splice variants in fractionated cell extracts. FS, fully spliced; ir1, intron 1 retention; ir1+2, retention of introns 1 and 2. Lower panel, polyacrylamide gel electrophoresis separation of protein extracts from the same cell fractionation. The cytoplasmic fraction is free of histone 3, and the nuclear fraction is free of the cytoplasmic isoform of fructose‐1,6‐bisphosphatase. T, total RNA/protein fraction; C, cytoplasmic RNA/protein fraction; N, nuclear RNA/protein fraction.

Many AS events either trigger nuclear retention (Gohring *et al*., 2014) or premature degradation of mRNAs via the nonsense mediated mRNA decay (NMD) pathway (Kalyna *et al*., 2012), and the affected mRNAs are unlikely to be translated. To test whether *CDKG1L* and *CDKG1S* mRNAs were affected by the NMD pathway, transcript accumulation in NMD defective lines *upf1‐5* and *upf3‐1* (Hori and Watanabe, 2005; Arciga‐Reyes *et al*., 2006) was compared with Col‐0 and neither transcript showed appreciable upregulation in these mutants, indicating that they are not targeted by NMD (Figure 3a).

To evaluate possible nuclear retention of the *CDKG1L* and *CDKG1S* transcripts, cell fractionation of Col‐0 protoplasts was followed by RT‐PCR to compare the cytoplasmic and nuclear fractions. Both *CDKG1L* and *CDKG1S* transcripts were exported to the cytoplasm, although to different extents (Figure 3b, top panel). Only the fully spliced form of a control transcript, SEF factor (AT5G37955), was found in the cytoplasm (Figure 3b, middle panel), as would be expected (Gohring *et al*., 2014). The purity of the fractions was confirmed by Western blot (Figure 3b, bottom panel).

These data suggested that both *CDKG1L* and *CDKG1S* transcripts are exported to the cytoplasm where they could be translated into proteins. Confirming that the short transcript can be translated, *CDKG1S* (AT5G63370.2) was found in the ribosomal fractions in RNA‐seq analyses (Aubry *et al*., 2014; Juntawong *et al*., 2014).

Taken together, our data suggest that CDKG1, CDKG2 and CYCL1 participate in a cascade of AS events that amplifies ambient temperature effects on target gene expression.

### CDKG1L and CDKG1S show distinct subcellular localization

To define the subcellular distribution of the two CDKG1 proteins *in planta*,* CDKG1L* and *CDKG1S* were transiently expressed in *Nicotiana benthamiana* leaf cells as cDNA‐GFP fusion proteins. We also generated a splicing‐competent version (*35S:CDKG1SC‐GFP*) consisting of *CDKG1* 5′UTR containing the full intron 1 region and the coding sequence that allows for the production of both *S* and *L* mRNA isoforms.

**Figure 4:**
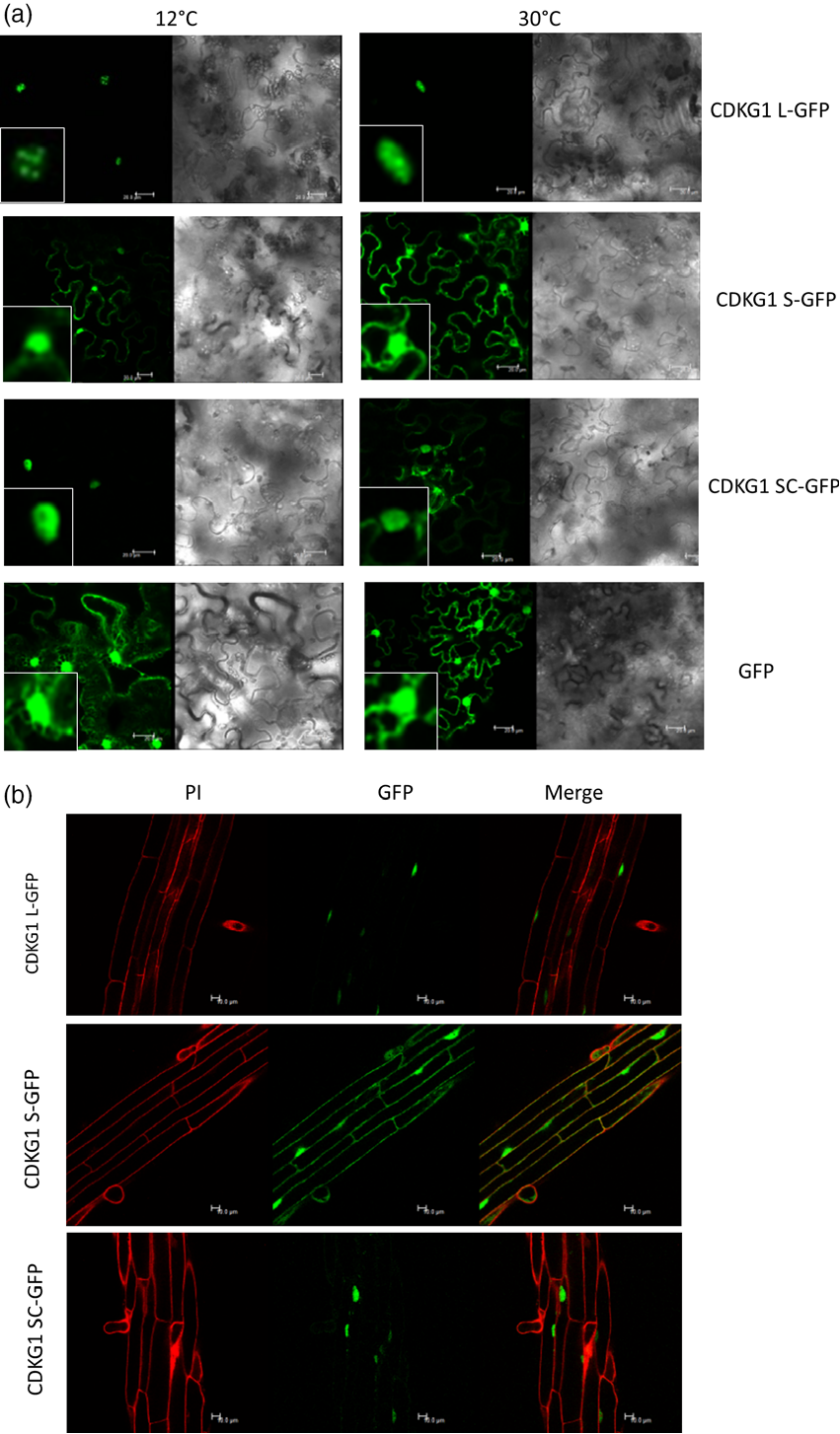
Effect of temperature on the localization of CDKG1‐GFP protein isoforms. (a) Subcellular localization of the different CDKG1 isoforms as C‐terminal GFP fusion proteins in *Nicotiana benthamiana* leaves. Infiltrated plants were kept at 12°C or 30°C as indicated for 48 h before leaves were imaged using confocal microscopy. Left‐hand‐side images GFP channel, right‐hand‐side brightfield images. Inset images show nucleus detail. (b) Localization of CDKG1‐GFP protein isoforms in Arabidopsis roots grown at 22°C. Roots from Arabidopsis plants stably expressing the different CDKG1 isoforms as GFP fusions as indicated were imaged using confocal microscopy. Cell walls were stained with propidium iodide (PI; red).

As previously reported (Huang *et al*., 2013), the CDKG1L‐GFP protein localized to the nucleus while CDKG1S, lacking the NLS, showed both nuclear and cytoplasmic GFP signal (Figure 4a). Moreover, CDKG1L‐GFP co‐localized strongly with the nuclear splicing factor RSp34 (Lorković *et al*., 2004a), and was associated with nuclear speckles (Figure S4a and b; Huang *et al*., 2013), as has been described for other spliceosome‐associated components (Lorković *et al*., 2004b).

Although temperature did not affect the cellular distribution of either CDKG1L or CDKG1S, CDKG1SC‐GFP‐containing cells showed a clear temperature‐dependent change in signal localization (Figure 4a): at low temperature, the GFP signal was detected only in the nucleus, while at higher temperatures GFP localized both to the cytoplasm and the nucleus (Figure 4a) as expected from the temperature‐dependent production of the CDKG1S transcript (described in Figure 2). The preferential localization of GFP signal to the cytoplasm at higher temperature was not due to the cleavage of the GFP moiety, as shown by Western blot (Figure S4c and d). We have also quantified relative nuclear to cytoplasm fluorescence in stably transformed lines (see below). There were no changes in the percentage of nuclear GFP fluorescence for CDKG1L‐GFP and for CDKG1S‐GFP, while for CDKG1SC‐GFP the ratio of nuclear to cytoplasmic fluorescence was lower at 27°C (Figure S5a), as expected for increased AS of the transcript. Although expression is driven by a strong promoter, these data together suggest that CDKG1 is not directly regulated by temperature at the protein level, and support the idea that temperature modulation acts on the transcript level, affecting the balance between *L* and *S* forms.

### Temperature‐dependent AS of *CDKG1* is necessary to compensate expression of *ATU2AF65A* along the temperature range

**Figure 5:**
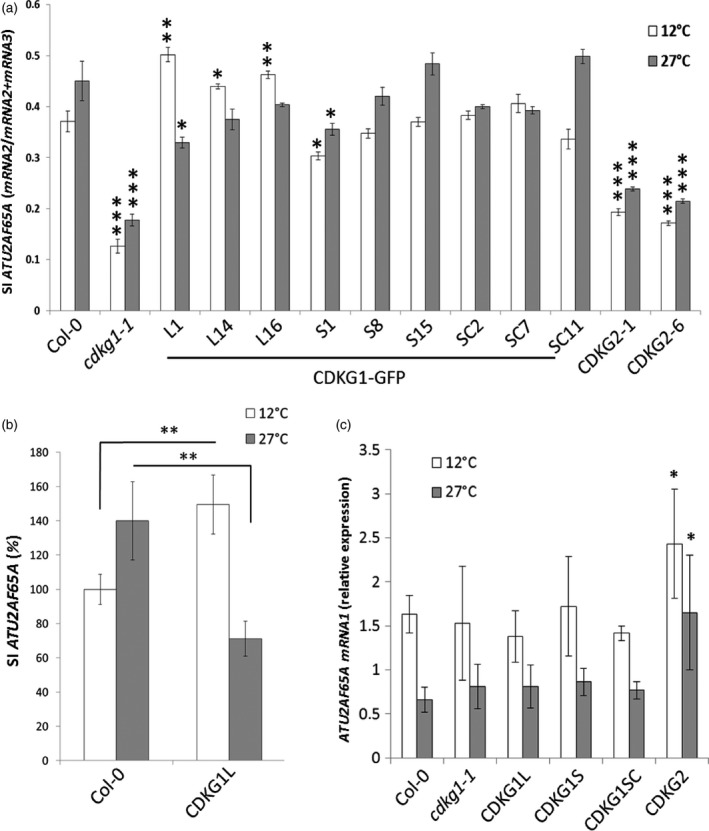
CDKG1 protein variants show temperature‐dependent effects on splicing. (a) Relative levels of *ATU2AF65A* estimated by reverse transcriptase‐quantitative polymerase chain reaction (RT‐qPCR). Splicing index (SI) represents the ratio mRNA2/[mRNA2 + mRNA3] in Col‐0 and transgenic lines in the *cdkg1‐1* background at 12°C or at 27°C. Three independent lines expressing the long (L), the short (S), or the splicing competent (SC) CDKG1‐GFP fusion constructs are shown. Data represent means ± SD (*n* ≥ 3). Overexpression of CDKG2‐GFP fusion showed no compensation of *ATU2AF65A* splicing pattern in two independent lines. Student's *t*‐test comparing the respective line with Col‐0 at the same temperature: ***P < 0.001; **P < 0.01; *P < 0.05. Primer position is reported on the respective splicing variant in Figure 1a. (b) Normalized levels of *ATU2AF65A* in Col‐0 and in the overexpression lines CDKG1L‐GFP. SI represents the ratio mRNA2/[mRNA2 + mRNA3]. Expression levels of Col‐0 at 12°C have been set to 100% and the other values been normalized accordingly for each line. Student's *t*‐test: **P < 0.01. (c) RT‐qPCR analysis of *ATU2AF65A* mRNA1 in Col‐0, *cdkg1‐1* and overexpression lines. The fully spliced mRNA1 levels are not significantly different between the control, the mutant and the CDKG1L, S or SC lines. CDKG2 overexpression lines show a statistically significant increase in mRNA1 level in comparison to Col‐0 at both temperatures. Data represent means ± SD (*n* = 3). *P < 0.05.

To test the functions of the different CDKG1 isoforms, *cdkg1‐1* mutants were stably transformed with the three different CDKG1‐GFP fusion constructs. As observed in transient assays, plants expressing CDKG1L showed exclusive nuclear GFP fluorescence, while CDKG1S‐GFP was found both in the nucleus and cytoplasm of root epidermal cells (Figure 4b). As for CDKG1SC‐GFP, we observed strong fluorescence in the nucleus with some faint cytoplasmic localization at normal growth temperature (22°C). To address whether CDKG1 variants could have different functions in splicing of *ATU2AF65A*, 2‐week‐old transgenic seedlings (*35S::CDKG1L‐GFP, 35S::CDKG1S‐GFP*,* 35S::CDKG1SC‐GFP*) were incubated at 12°C or 27°C for 48 h prior to RNA isolation. As previously observed, *ATU2AF65A* SI increased in Col‐0 control plants from 12°C to 27°C in RT‐qPCR (Figures 1d and 5a).

The full‐length CDKG1L could restore mRNA processing of *ATU2AF65A* at low temperatures but, surprisingly, showed severely reduced efficiency at high temperatures (Figure 5a). In relative levels, we calculated that *ATU2AF65A* splicing in CDKG1L‐GFP‐expressing plants was severely reduced by the temperature shift from 12°C to 27°C (0.65‐fold; Figure 5b). While the efficiency of splicing intron 11 was also reduced in comparison to Col‐0 at 27°C (0.67‐fold), at low temperature all CDKG1L lines had somewhat higher splicing compensation than the control (1.2‐fold; Figure 5b). Expression of CDKG2‐GFP could not restore the splicing of *ATU2AF65A* in the *cdkg1* mutant line (Figure 5a), but did cause a small increase in the fully spliced *ATU2AF65A* mRNA1 (Figure 5c).

In contrast, the splice‐competent variant CDKG1SC‐GFP was able to rescue the *ATU2AF65A* splicing phenotype at both temperatures, and a similar result was observed for the lines expressing CDKG1S‐GFP (Figure 5a).

**Figure 6:**
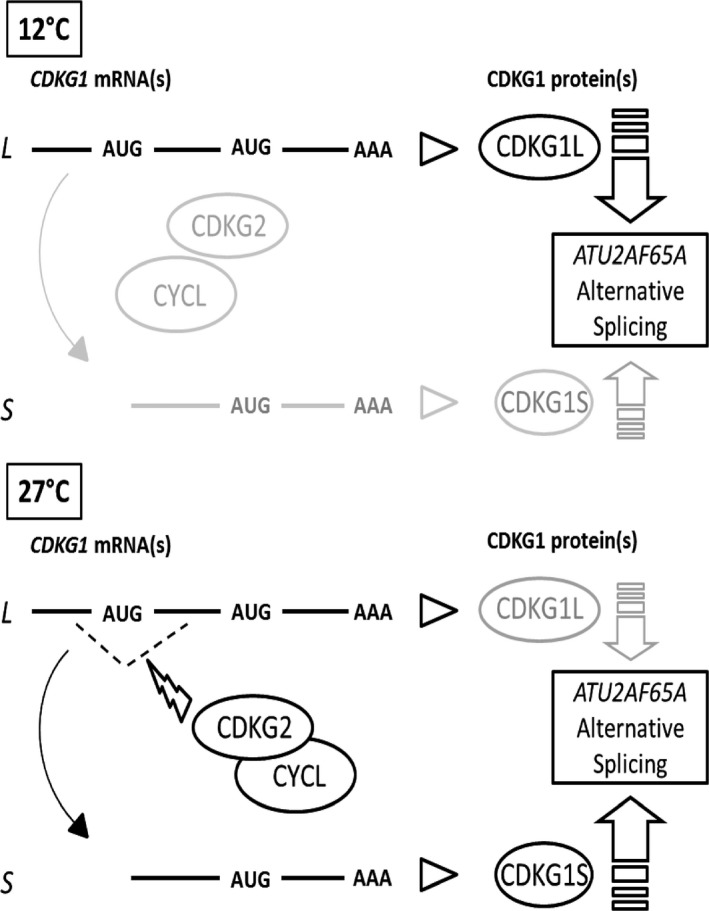
Proposed model for the temperature‐dependent splicing cascade regulating *ATU2AF65A*. At low temperature, *CDKG1* splicing is inhibited and translation of the full‐length mRNA (*L*) results mainly in CDKG1L. At high temperature, the CDKG2‐CYCL1 complex activates *CDKG1* splicing, and in turn CDKG1S protein is translated to compensate for the loss of efficiency of CDKG1L on the alternative splicing (AS) of *ATU2AF65A*. The associated splicing factors interacting with CDKG and the mRNAs were omitted for simplicity.

These data show that splicing of CDKG1 is necessary to maintain the correct balance between the *ATU2AF65A* splice forms across the temperature range (Figure 6).

## Discussion

We report a temperature‐sensing module involving CDKG1, CDKG2 and CYCL1 that transduces ambient temperature information to downstream targets by modulation of AS (Figure 6). In particular, this module impinges on the AS of *ATU2AF65A*, a splicing factor already known to be environmentally sensitive (Petrillo *et al*., 2014; Verhage *et al*., 2017), and thus provides a mechanism to integrate temperature variation with the gene expression machinery.

Splicing is a conceptually attractive mechanism for integrating the thermal response in a flexible and sensitive manner. The spliceosome is one of the largest cell molecular complexes and its temperature dependency has been widely documented (Reddy and Shad Ali, 2011; Staiger and Brown, 2013; Streitner *et al*., 2013; Schlaen *et al*., 2015; Verhage *et al*., 2017). The RNA secondary structures required for splicing are inherently thermo‐sensitive, and could be tuned either on the individual gene basis for specific responses or could even affect gene expression on a genome‐wide scale. A mechanism based on this general principle operates during the heat shock response in budding yeast *Saccharomyces cerevisae* (Meyer *et al*., 2011) and on the thermal‐sensitive splicing of a clock gene, *period‐3*, in *Drosophila melanogaster*, potentially providing an adaptive mechanism across different climatic zones (Low *et al*., 2008). In plants, the spliceosome itself may be directly involved mediating temperature responses as the LSM2‐8 complex, the protein moiety of the U6 snRNP, is required in Arabidopsis to correctly couple spliceosome activity to environmental conditions, including temperature (Carrasco‐López *et al*., 2017). Our results indicate that protein kinases associated with the spliceosome are capable of modulating its activity, providing an additional route for environmental information to affect gene expression. It will be interesting to determine whether this is a widespread mechanism across species.

Severe temperature changes clearly affect AS, but the thermodynamic effects of ambient temperature fluctuations are more subtle and likely to require augmentation by associated factors.

Plants have several classes of splicing factors, and some exist as multigene families that may help maintain transcript homeostasis across environmentally relevant temperatures (Wang *et al*., 2012; Schlaen *et al*., 2015; Marshall *et al*., 2016) and as well as in more extreme conditions (Ciuzan *et al*., 2015; Kim *et al*., 2017). Ambient temperature variations are also known to alter the availability of specific splicing factors in grape leaves where their abundance correlates with the level of AS (Jiang *et al*., 2017). Moreover, splicing factors are subjected to various forms of post‐translational modification that could modulate splice site selection. Protein phosphorylation is one of these modifications, and the LAMMER kinase (Savaldi‐Goldstein *et al*., 2000) and CDKG1 (Huang *et al*., 2013; Zheng *et al*., 2014) are proposed to have this activity in plants.

Based on our results, CDKG1 is required for the AS of *ATU2AF65A* across the tested temperature range, contrary to its reported role in meiosis where it is only required at high ambient temperature (Zheng *et al*., 2014). It will be interesting to determine if the meiosis specific effect is through the regulation of AS. Loss of ATU2AF65A affects flowering time at high and low temperatures (Verhage *et al*., 2017), and this combinatorial cascade composed of protein kinases and AS could provide a sensitive means of transducing ambient temperature or other signals into altered gene expression and growth, providing an interesting paradigm to regulate plant development in response to environmental changes.

CDKG2 and CDKG1 are not functionally redundant with regard to mRNA splicing, although they may occasionally affect the same target as CDKG2 increases the level of fully spliced *ATU2AF65A* mRNA1. While relative expression does not change along the temperature gradient, *CDKG1* is alternatively spliced in a temperature‐sensitive manner – as has also been observed for other splicing‐related genes (Reddy and Shad Ali, 2011; Verhage *et al*., 2017). *CDKG1* probably originated from a retrotransposition event early in the Arabidopsis lineage with the loss of the original intron sequences (Zhang, 2005). However, *CDKG1* still carries memories of splice sites and exonic enhancer sequences that enable coding and non‐coding sequences to be spliced out, an event that was termed exitron (exonic intron) splicing if occurring in coding regions (Marquez *et al*., 2015). The fact that splicing events are still present in the retrotransposed *CDKG1* gene suggests a strong evolutionary pressure on these splicing events and underscores the importance of post‐transcriptional modifications for this gene. Particularly, the presence of the first intron sequence is essential for proper expression and subcellular location of *CDKG1* variants along the temperature range as well as for the processing of a downstream target. Our hypothesis was supported by the fact that while *ATU2AF65A* intron 11 was inefficiently removed in the presence of the full‐length CDKG1L at high temperature, both the short CDKG1S and the splicing competent CDKG1SC were able to complement splicing.

The role of this group of CDKs in AS could be evolutionarily conserved across animals and plants, at least partially. The large isoform of mammalian CDK11, p110 activates while the short isoform represses splicing/AS in tissue culture cells (Loyer *et al*., 2008). While the C‐terminal kinase domain is highly conserved between CDKG and CDK11, the large N‐terminal domain in plant proteins suggests functional divergence. This divergence may involve ambient temperature sensing as CDKG1S and CDKG1L proteins also differ in their N‐terminal domains. Ambient temperature sensing/response is of particular interest in terms of organisms that do not regulate their body temperature. A phylogenic functional comparison CDK11/CDKG function in poikilotherms as compared with homeotherms could be informative as how gene expression networks adapt to ambient temperature.

In addition, the temperature‐dependent AS splicing of CDKG1 has wider implications in terms of genomic recombination and stability. CDKG1 was shown to be involved in chromosome pairing and recombination during meiosis at high ambient temperature (Zheng *et al*., 2014). CDKG1 has previously been shown to be involved in the splicing of the *CalS5* gene in the tapetal cells affecting pollen fertility (Huang *et al*., 2013). In addition, a recent survey of transcripts in the male sexual lineage has shown that mis‐splicing of the MPS1 gene leads to meiotic defects (Walker *et al*., 2018). Taken together, these observations suggest that splicing is an important regulatory mechanism during meiotic cell divisions. Temperature‐dependent splicing could, at least in part, explain the temperature sensitivity of the male germ line. Therefore, a detailed characterization of AS in meiocytes along the temperature range could be informative.

Our data support a model where *CDKG1* mRNA acts as a thermo‐switch that is activated by CDKG2 and CYCL1 (Figure 6). The mechanisms and the pattern of interactions remain to be determined, but our data suggest a clear, previously unidentified link between temperature, CDKs and AS. In addition, the observation that CDKG1 is itself regulated at the mRNA level by both temperature and the CDKG2‐CYCL1 complex reveals a multi‐layered AS‐kinase cascade.

## Experimental procedures

### Plant material and growth conditions

The wild‐type Columbia (Col‐0) and mutant stocks used in this study were obtained from the Nottingham Arabidopsis Stock Centre. The *cdkg1‐1*,* cdkg2‐2*,* cycL1‐1*,* upf1‐5* and *upf3‐1* mutant lines have been previously described (Hori and Watanabe, 2005; Arciga‐Reyes *et al*., 2006; Zheng *et al*., 2014; Ma *et al*., 2015). Moist sterilized seeds were pre‐treated for 3 days at 4°C in the dark and germinated on Petri‐plates. Plants transferred to soil after 1 week or sown direct to soil were grown in standard greenhouse conditions at 22°C supplemented with 16 h light and 8 h dark. For analysis of *ATU2AF65A* splicing plants were grown in plant medium plates (0.5 × MS salts and vitamins, pH 5.8, 0.7% plant agar) for 2 weeks at 22°C in constant light conditions to minimize the reported light effect on *ATU2AF65A* splicing (Petrillo *et al*., 2014). Plants were transferred to 12°C or 27°C for 2 days and collected for mRNA.

### Construct generation and plant transformation

*CDKG1SC*,* CDKG1L*,* CDKG1S* and *CDKG2* sequences were cloned in frame with GFP sequence at the 3′‐end by fusion PCR. The entire cassette was cloned into the pB7WG2,0 (Karimi *et al*., 2002) destination vector by Gateway™ cloning and transformed into *Agrobacterium tumefaciens* strain GV3101 by electroporation. Arabidopsis *cdkg1‐1* plants were transformed by floral dip (Clough and Bent, 1998). Single‐insertion, BASTA‐resistant plants were selected, and a minimum of three lines per construct were analysed.

For transient expression in *N. benthamiana* leaves, the *CDKG1x‐GFP* cassette was cloned into the pEAQ‐HT‐DEST2 vector transformed into *A. tumefaciens* LBA4404 strain. Leaf infiltration was performed as described (Sainsbury *et al*., 2009). After 5 days, leaves were harvested for confocal imaging of GFP or for protein extraction.

### RNA extraction, RT and qPCR

Total RNA was extracted from whole rosettes using the RNeasy Plant Mini kit (Qiagen); 1 μg total mRNA was used to generate cDNA using the SuperScript^®^ III First‐strand Synthesis kit (Invitrogen); 50 ng cDNA was used for the PCR reaction. The primers used for the analysis of the AS of the different genes are listed in Table S3. qPCRs were performed using the LightCycler 480 (Roche). Typically 10 ng of cDNA was used in a 20 μl reaction containing 0.25 μm of each primer and 10 μl LightCycler^®^ 480 SYBR Green I Master (Roche). Each sample was done in triplicate, and expression of *PP2AA3* (AT1G13320) was used as a reference (Czechowski, 2005). Data were analysed using the LightCycler^®^ 480 Software (Roche).

### Protoplast isolation and subsequent cell fractionation

Mesophyll protoplasts were isolated from 3‐week‐old Col‐0 plants as described by Wu *et al*. (2009). Subsequent cell fractions were prepared as described by Gohring *et al*. (2014) with slight modifications. Briefly, 2 × 10^6^ *Arabidopsis thaliana* mesophyll protoplasts were resuspended in 1 ml NIB lysis buffer [10 mm MES‐KOH pH 5.5, 200 mm sucrose, 2.5 mm EDTA, 2.5 mm dithiothreitol (DTT), 0.1 mm spermine, 10 mm NaCl, 0.2% Triton X‐100, 1 U μl^−1^ RNasin (Promega)] and subsequently lysed using a 25‐G gauge needle (6–10 passages). Complete lysis was confirmed by light microscopy. For the total fraction, 100 μl of lysed cells was immediately resuspended in 1 ml TRIzol (Ambion) and kept on ice until the remaining fractions were processed. The lysate was pelleted for 10 min at 500 ***g***, and 1 ml of supernatant, which represents the cytoplasmic fraction, was removed and centrifuged for another 15 min at 10.000 ***g***. Then, 800 μl of supernatant was resuspended in 8 ml TRIzol and the pellet, which represents the nuclear fraction, resuspended in 4 ml NRBT (20 mm Tris‐HCl pH 7.5, 25% glycerol, 2.5 mm MgCl_2_, 0.2% Triton X‐100), centrifuged at 500 ***g*** for 10 min and washed three times. After washing, the nuclear pellet was resuspended in 500 μl NRB2 (20 mm Tris‐HCl pH 7.5, 250 mm sucrose, 10 mm MgCl_2_, 0.5% Triton X‐100, 5 mm β‐mercaptoethanol) and carefully overlaid on top of 500 μl NRB3 (20 mm Tris‐HCl pH 7.5, 1.7 m sucrose, 10 mm MgCl_2_, 0.5% Triton X‐100, 5 mm β‐mercaptoethanol) and centrifuged at 16.000 ***g*** for 45 min. Finally, the nuclear pellet was resuspended in 1 ml TRIzol and RNA as well as proteins of all fractions were isolated following the manufacturer's instructions. Western blot analyses (see below for details) using anti‐H3 and anti‐FBPase (1:5000, Agrisera) antibodies were performed to confirm purity of nuclear and cytoplasmic fractions, respectively.

### Protein extraction and Western blotting

Total proteins were extracted in RIPA buffer (1 mm sodium phosphate buffer pH 7, 150 mm NaCl, 2 mm EDTA, 1% Triton X‐100, 0.1% sodium dodecyl sulphate, 1 mm DTT and protease inhibitors cocktail). The extract was filtrated through a Celtrix 30‐μm filter (Partec) and used for immunoprecipitation using the μMACS GFP Protein isolation kit (Miltenyi Biotec GmbH) following the manufacturer's instructions. For *in vitro* expression, proteins were produced in the T7‐TNT Quick coupled transcription/translation system using wheat germ extract according to the manufacturer's instructions (Promega). Proteins were separated in 10% mini‐Protean TGX precast gels (Biorad). Western blot was done using anti‐GFP monoclonal antibody (Santa Cruz) diluted 1:2500 and horseradish peroxidase (HRP)‐conjugated anti‐mouse antibody (Sigma, 1:10 000). Detection was done using ECL Select Western Blot Detection Reagent (Amersham) and visualized using Image Quant LAS 4000 (GE).

For the cell fractionation experiments, proteins were separated using 10–16% polyacrylamide gels and the Xcell SureLock Mini cell system (LifeTechnologies), and transferred to PVDF membranes. The primary antibodies used were anti‐H3 or anti‐FBPase (Agrisera) both at a dilution of 1:5000, and the secondary antibody used was rat anti‐rabbit IgG coupled to HRP (Cell Signaling Technology) at a 1:10 000 dilution.

Detection was performed using the ECL Western Blotting Detection Reagent (Amersham), and membranes exposed to CL‐XPosure films and developed using a Curix60 (AGFA) developer.

### Microscopy

Roots stained with propidium iodine or *N. benthamiana* infiltrated leaves were imaged using a Leica TCS SP5 II confocal laser‐scanning microscope controlled by Leica LAS‐AF software. Multiple plants per line and multiple lines were observed, and representative images are shown.

GFP levels in cells were quantified according to McCloy *et al*. (2014) with slight modifications. Briefly, a single in‐focus plane was acquired. Using ImageJ (v1.51, NIH), an outline was drawn around the sample area and the mean fluorescence was measured, while visible nuclei were selected independently and GFP signal calculated as the sum of all the nuclei intensity.

### Statistical analysis

Statistical analyses were performed using PRISM 6.0 (GraphPad Software, La Jolla) or Excel (Microsoft Office, Microsoft). *P*‐values were calculated using an unpaired, two‐legged Student's *t*‐test (***P < 0.001; **P < 0.01; *P < 0.05; ns, not significant). Data represent means ± standard deviation (*n* ≥ 3).

## Author's contributions

NC, CN, JHD and AB conceived the project and designed research; NC, CN, AF and DD performed research; NC and CN analysed data; NC, CN, JHD and AB wrote the paper.

## Conflict of interest

The authors declare no conflict of interest.

## Supporting information


**Figure S1.** RT‐qPCR analysis of AT*U2AF65A* splice variants in Col‐0 and *cdkg1‐1* mutant.
**Figure S2.** Analysis of CDKG1 AS.
**Figure S3.** Gel separation of RT‐PCR products of *CDKG2* and *CYCLIN L1* splice variants.
**Figure S4.** Analysis of CDKG1‐GFP protein expression.
**Figure S5.** Quantification of the GFP signal in 35S‐CDKG1 lines.

**Table S1.** List of the genes investigated in Col‐0 and *cdkg1‐1* mutant lines.
**Table S2.** List of the transcripts and intron splicing events investigated.
**Table S3.** List of RT‐PCR, RT‐qPCR and cloning primers used.
