# Supplementary figures and images for: The cyclin‐dependent kinase G group defines a thermo‐sensitive alternative splicing circuit modulating the expression of Arabidopsis ATU2AF65A
Source: Plant J. 2018 May 10;94(6):1010–22. doi: 10.1111/tpj.13914 (PMC6032924; doi:10.1111/tpj.13914)

**SUPPORTING FIGURES**

**
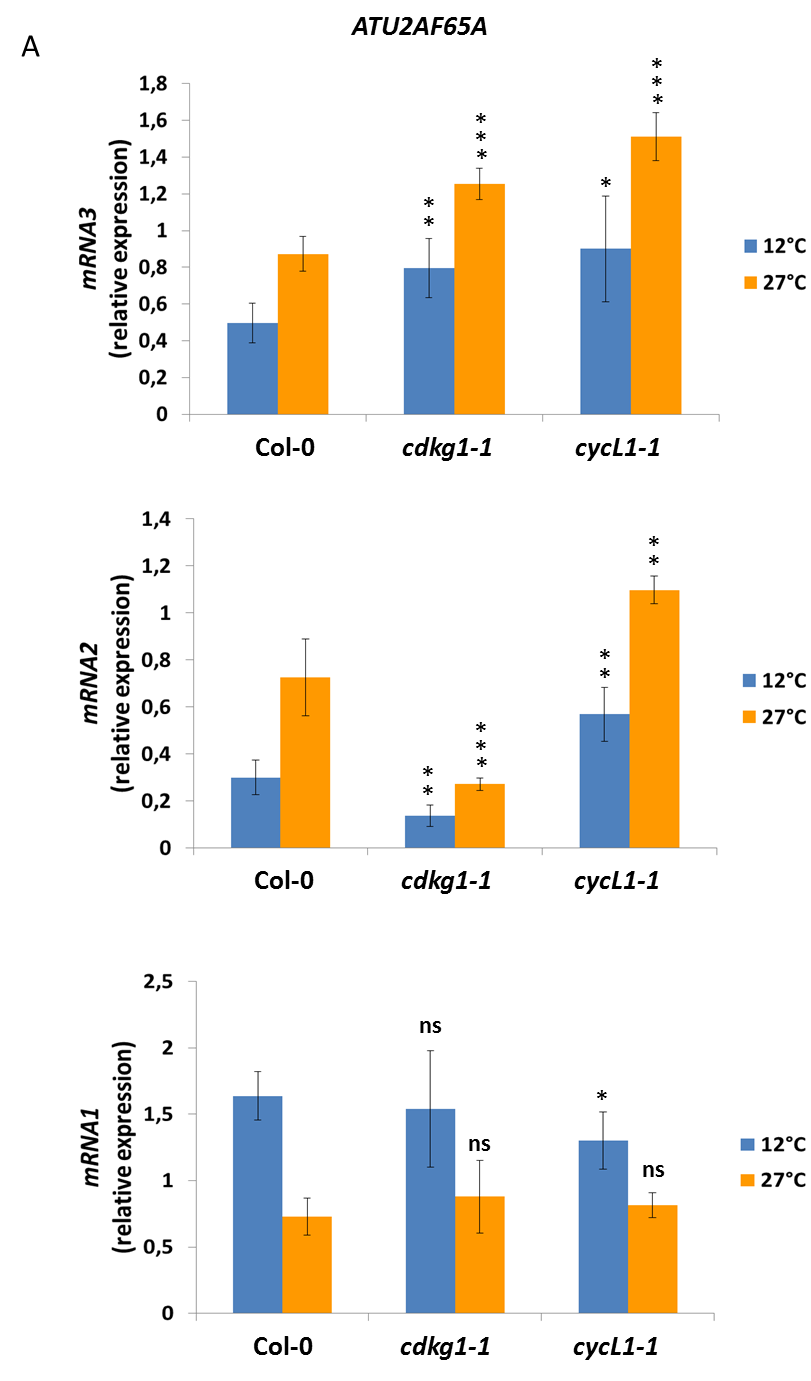
**

**Figure S1**


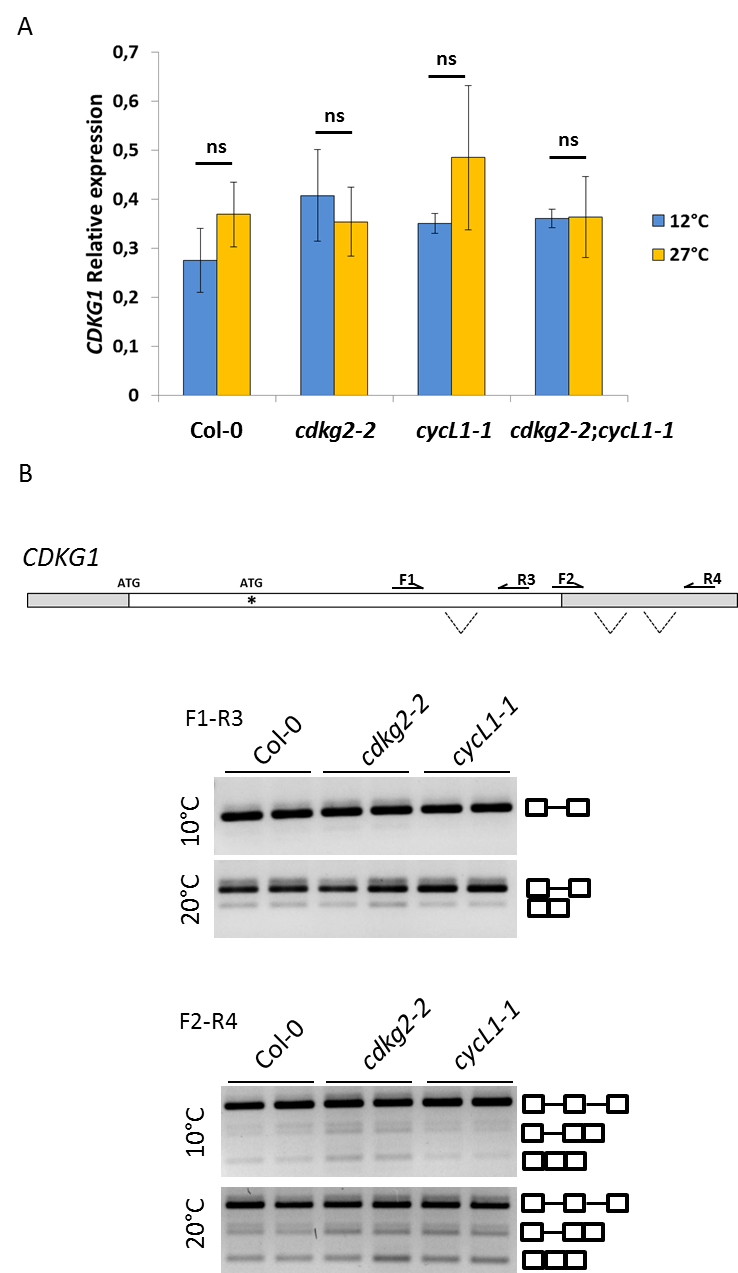


**Figure S2**


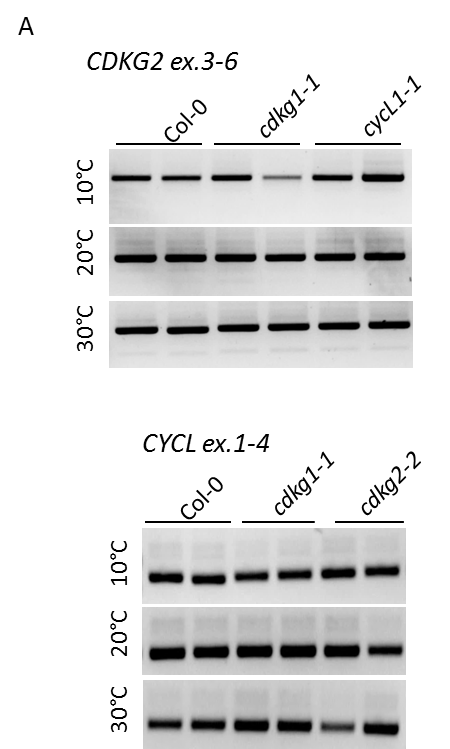


**Figure S3**


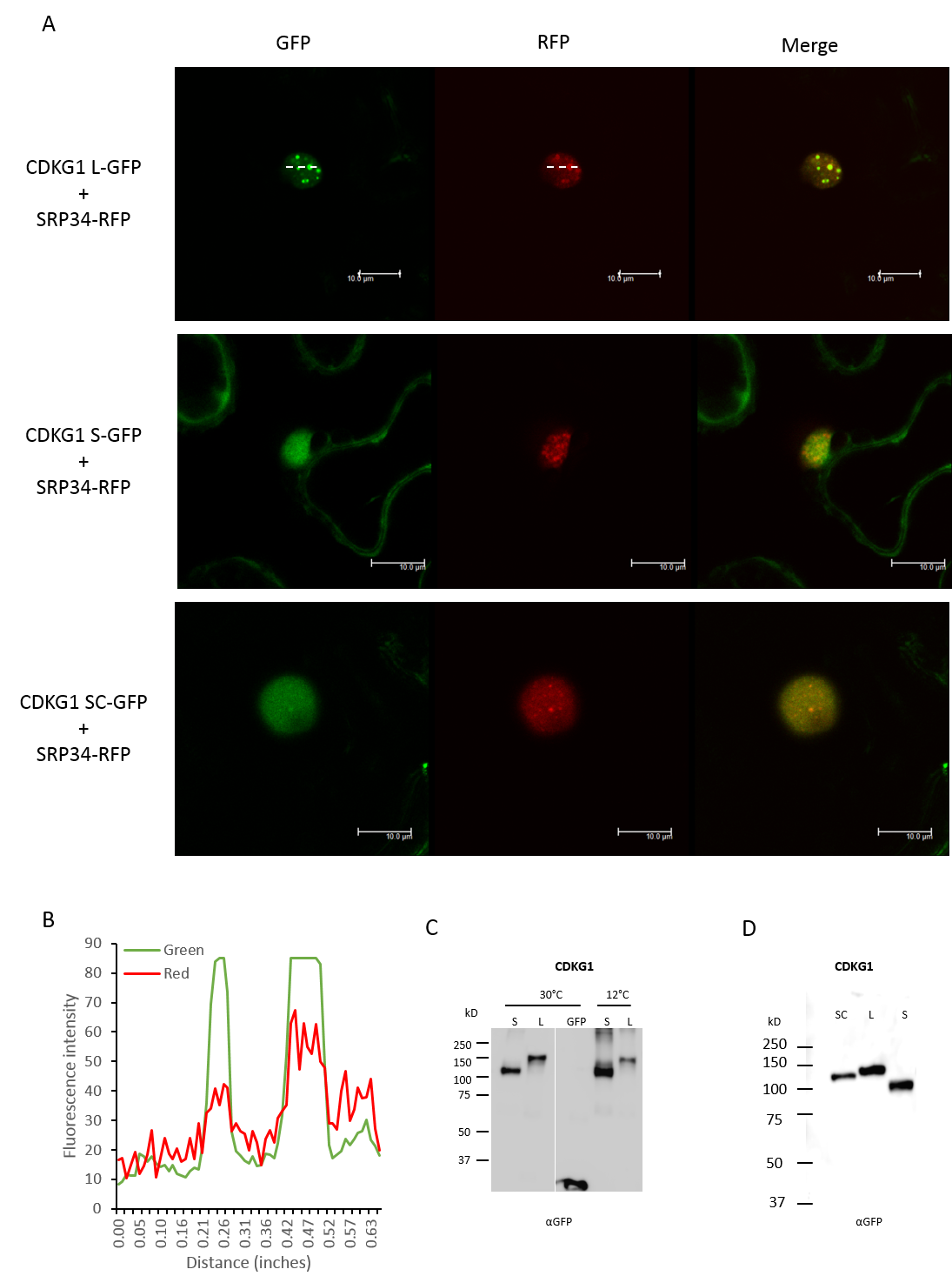
**Figure S4**


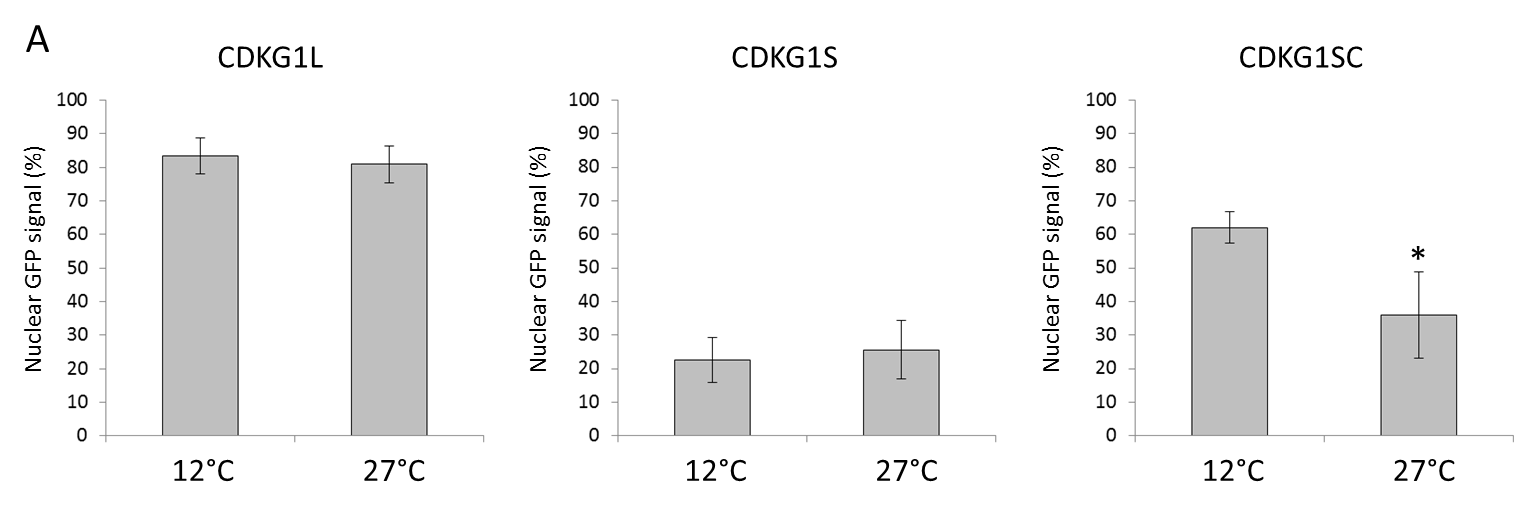


**Figure S5**

Supplement: Supplementary file 1 — Figure S1. RT‐qPCR analysis of ATU2AF65A splice variants in Col‐0 and cdkg1‐1 mutant. Figure S2. Analysis of CDKG1 AS. Figure S3. Gel separation of RT‐PCR products of CDKG2 and CYCLIN L1 splice variants. Figure S4. Analysis of CDKG1‐GFP protein expression. Figure S5. Quantification of the GFP signal in 35S‐CDKG1 lines. [file TPJ-94-1010-s001.docx]
