# Supplementary material for: The cyclin‐dependent kinase G group defines a thermo‐sensitive alternative splicing circuit modulating the expression of Arabidopsis ATU2AF65A
Source: Plant J. 2018 May 10;94(6):1010–22. doi: 10.1111/tpj.13914 (PMC6032924; doi:10.1111/tpj.13914)
Supplement: Supplementary file 2 — Table S1. List of the genes investigated in Col‐0 and cdkg1‐1 mutant lines. Table S2. List of the transcripts and intron splicing events investigated. Table S3. List of RT‐PCR, RT‐qPCR and cloning primers used. [file TPJ-94-1010-s002.docx]

**SUPPORTING TABLES**

**Table S1.** List of the genes investigated by RT-PCR in Col-0 and *cdkg1-1* mutant lines.

| **Gene name** | **Gene ID** | **Gene name** | **Gene ID** | **Gene name** | **Gene ID** | **Gene name** | **Gene ID** |
| --- | --- | --- | --- | --- | --- | --- | --- |
| *ATSRP30* | AT1G09140 | *ATSRP41* | AT5G52040 | *ATRS2Z32* | AT3G53500 | *CCA1* | AT2G46830 |
| *ATSRP31* | AT3G61860 | *ATSC35* | AT5G64200 | *ATRS2Z33* | AT2G37340 | *TOC1* | AT5G61380 |
| *ATRSP31A* | AT2G46610 | *ATSCL28* | AT5G18810 | *PTB1* | AT3G01150 | *PRR7* | AT5G02810 |
| *ATSRP34* | AT1G02840 | *ATSCL30* | AT3G55460 | *U2AF65A* | AT4G36690 | *GRP7* | AT2G21660 |
| *ATSRP34A* | AT3G49430 | *ATSCL30A* | AT3G13570 | *U2AF65B* | AT1G60900 | *CDKG1* | AT5G63370 |
| *ATRSP34B* | AT4G02430 | *ATSCL33* | AT1G55310 | *ATSR45* | AT1G16610 | *CDKG2* | AT5G63370 |
| *ATSRP40* | AT4G25500 | *ATRSZ22* | AT4G31580 | *CAT2* | AT4G35090 | *CYCL1* | AT2G26430 |

**Table S2.** List of the transcripts and intron splicing events investigated by RT-PCR or by qRT-PCR in this study.

| **Gene name** | **Transcript ID** | **Intron coordinates** | **Intron size (bp)** | **Alternative splicing event** | **Reported name** |
| --- | --- | --- | --- | --- | --- |
| *ATU2AF65A* | AT4G36690.1 | chr4:17,294,703-17,294,431 | 273 | Intron splicing | mRNA1 |
| *ATU2AF65A* | AT4G36690.3 | chr4:17,294,703-17,294,604 | 100 | Alternative 3'ss | mRNA2 |
| *ATU2AF65A* | AT4G36690.2 | chr4:17,294,703-17,294,431 | 273 | Intron retention | mRNA3 |
| *CDKG1* | AT5G63370.1 | chr5:25,387,099-25,386,783 | 317 | Intron retention | *CDKG1L* |
| *CDKG1* | AT5G63370.2 | chr5:25,387,099-25,386,783 | 317 | Intron splicing | *CDKG1S* |
| *CDKG1* | AT5G63370.5 | chr5:25,385,150-25,385,078 | 73 | Intron splicing |  |
| *CDKG1* | AT5G63370.6 | chr5:25,384,506-25,384,403 | 104 | Intron splicing |  |
| *CDKG1* | AT5G63370.7 | chr5:25,384,359-25,384,222 | 138 | Intron splicing |  |
| *CDKG2* | AT1G67580.2 | chr1:25,327,623-25,327,512 | 112 | Intron splicing |  |
| *CYCL1* | AT2G26430.1 | chr2:11,245,352-11,245,269 | 84 | Intron splicing |  |
| *CYCL1* | AT2G26430.2 | chr2:11,245,352-11,245,262 | 91 | Alternative 3'ss |  |

**Table S3. List of RT-PCR, RT-qPCR and cloning primers used.**

| **Primer name** | **Sequence** | **Application** |
| --- | --- | --- |
| ATU2AF65A_F | GCACAGCAGCAAATAGCTT | RT-PCR/qPCR of U2AF65A |
| ATU2AF65A_R | GGCCTGCCACTGGCTCACCATTGG | RT-PCR of U2AF65A |
| ATU2AF65A_R3 | GCGCAATTAAGAGGTCTCTT | qPCR of U2AF65A |
| ATU2AF65A_R2 | TTCTTTATAGCAAAAGGCAAACTT | qPCR of U2AF65A |
| ATU2AF65A_R1 | ATTGGTCAAAGCACCAAACTTT | qPCR of U2AF65A |
| PP2A-FW | TAACGTGGCCAAAATGATGC | Reference gene for qPCR |
| PP2A-RV | GTTCTCCACAACCGCTTGGT | Reference gene for qPCR |
| CDKG1_F | CGCGTCTCCGAGAGGAAAA | RT-PCR/qPCR of CDKG1 |
| CDKG1_R | TTTGACGCCTCTCGTCATCT | RT-PCR of CDKG1 |
| CDKG1_qF | GCAGTGGACATCTCAGCGTA | qPCR of CDKG1 |
| CDKG1_qR | TCACAGACCTGCTCCCAAAC | qPCR of CDKG1 |
| CDKG1_R1 | TCCGATTCACAAGTACCGGAAG | qPCR of CDKG1 |
| CDKG1_R2 | CATCAACACCCCCTGCGGAC | qPCR of CDKG1 |
| CDKG1_F1 | GTTCCCGGGTAAGAGTGAGC | RT-PCR of CDKG1 |
| CDKG1_R3 | CGCTTTGGAGGATATGTCGG | RT-PCR of CDKG1 |
| CDKG1_F2 | ATCACAAACGTGAGAATGGA | RT-PCR of CDKG1 |
| CDKG1_R4 | TTATTGAAATGAACTTGATTTAGCTTAG | RT-PCR of CDKG1 |
| GFP qPCR Fw | TGGTCCTGCTGGAGTTCG | qPCR of GFP |
| GFP qPCR Rv | CTTGTACAGCTCGTCCATGC | qPCR of GFP |
| CDKG2_3F | TGGCCTGGGTTCTCTAAACT | RT-PCR of CDKG2 |
| CDKG2_6R | ACAAGCACTGTCAGAAAGCC | RT-PCR of CDKG2 |
| CYCL1_1F | ATTTCCCGATTTCGTCGCCT | RT-PCR of CYCL1 |
| CycL_4R | AAGTTCTCCCTGCGACACTC | RT-PCR of CYCL1 |
| AtCDKG1L attB1 | GGGGACAAGTTTGTACAAAAAAGCAGGCTCGATGGCAGCAGGGGGTGTTGATG | Cloning |
| AtCDKG1S attB1 | GGGGACAAGTTTGTACAAAAAAGCAGGCTTGATGTCGCCAGAACCTAGTTATC | Cloning |
| AtCDKG1SC attB1 | GGGGACAAGTTTGTACAAAAAAGCAGGCTTGCGC GTC TCC GAG AGG AAA ATT TAG | Cloning |
| AtCDKG2 attB1 | GGGGACAAGTTTGTACAAAAAAGCAGGCTTGATGGCGGCTGGGAGGAATATAAG | Cloning |
| CDKG1-GFP-F | TATCCTCCAAAGCGGGGAGGTGGTGGCGGCCGCATGGTGAGCAAGG | Cloning |
| CDKG2-GFP-F | GGCGGTCTGTTTGGCGGAGGTGGTGGCGGCCGCATGGTGAGCAAGG | Cloning |
| GFP cter attB2 | GGGGACCACTTTGTACAAGAAAGCTGGGTTTTA CTT GTA CAG CTC GTC CAT GC | Cloning |
