# Supplementary material for: The cyclin‐dependent kinase G group defines a thermo‐sensitive alternative splicing circuit modulating the expression of Arabidopsis ATU2AF65A
Source: Plant J. 2018 May 10;94(6):1010–22. doi: 10.1111/tpj.13914 (PMC6032924; doi:10.1111/tpj.13914)
Supplement: Supplementary file 3 [file TPJ-94-1010-s003.docx]

**Supporting information legends**

**Table S1.** List of the genes investigated by RT-PCR in Col-0 and *cdkg1-1* mutant lines.

**Table S2.** List of the transcripts and intron splicing events investigated by RT-PCR or by qRT-PCR in this study.

**Table S3.** List of RT-PCR, RT-qPCR and cloning primers used.

**Figure S1.** RT-qPCR analysis of AT*U2AF65A* splice variants in Col-0, *cdkg1-1* and *cycL1-1* mutants. (*A*)The fully spliced mRNA1 levels are not significantly different between the *cdkg1-1* mutant and the Col-0 control (lower panel) while mRNA2 and mRNA3 levels (middle and upper panels) are decreased or increased respectively. In *cycL1-1*, both mRNA2 and mRNA3 expression is increased while mRNA1 levels are slightly decreased at 12°C in comparison to Col-0. Primer position is reported on the respective splicing variant in Fig.1*A* Data are means ± SD (n≥3) Student’s t-test comparing Col-0 to *cdkg1-1* at the respective temperature: ***p < 0.001; **p < 0.01; *p < 0.05; ns, not significant.

**Figure S2.** Analysis of CDKG1 alternative splicing. (*A*) Quantification of *CDKG1* total mRNA levels estimated by RT-qPCR in Col-0 and in mutant seedlings. 15-day-old seedlings were incubated for 2 days at 12°C (blue) or at 27°C (orange) before mRNA extraction. Data represent means ± SD (n = 3). Student’s t-test comparing 12°C to 27°C: p > 0.05; ns, not significant. (*B*) Upper panel. Schematic representation of *CDKG1* mRNA. White and grey boxes correspond to coding and non-coding sequences (UTRs) respectively. The position of the start codons (ATG) are indicated. Dotted lines represent alternative intron splicing events investigated by RT-PCR. Position of primers used is reported. Lower panels, RT-PCR products after gel separation showing alternative *CDKG1* intron splicing pattern in Col-0 and mutant backgrounds. The reported primer pairs above the picture denote the amplified fragment. White boxes and black line on the right schematize the splicing of the alternative intron(s) according to the associated product in the gel picture.

**Figure S3.** Gel separation of RT-PCR products of *CDKG2* and *CYCLIN L1* splice variants. (*A*)Two-week old seedlings were incubated for two days at the indicated temperature before mRNA extraction. RT-PCR analysis did not show difference in alternative splicing of the target genes along the temperature shift or in the different backgrounds (reported above). The exons spanning the amplified fragments are indicated.

**Figure S4.** Analysis of CDKG1-GFP protein expression. (*A*) CDKG1 proteins co-localise with the spliceosome component SRP34 in *N. benthamiana* leaves. The green channel (GFP) shows the localisation of the different CDKG1-GFP protein isoforms as indicated, the red (RFP) channel the localization of the SRP34-RFP protein, BF in a bright field image of the leave region and merge an overlay of the three channels. (*B*) Fluorescence intensity plot showing co-localisation of the green and red signals across the dotted line depicted in (*A*). (*C*) Western blot analysis of CDKG1-GFP proteins expressed in infiltrated *Nicotiana benthamiana* leaves incubated at either 12$^{\circ}$C or 30$^{\circ}$C for 48h before protein extraction. Total proteins were separated in 10% SDS-PAGE gels and the blots probed with anti-GFP antibody. (*D*) Western blot analysis of CDKG1-GFP proteins expressed in an *in vitro* translation system. Proteins were separated in 10% SDS-PAGE gels and the blots probed with anti-GFP antibody.

**Figure S5.** Quantification of the GFP signal in 35S-CDKG1 lines. (*A*) The GFP intensity for each transgenic line (CDKG1L, CDKG1S and CDKG1L) was measured in unaltered root cells and expressed as percentage of the nuclear signal over the total sample fluorescence. Data represent means ± SD (n ≥ 3); *p < 0.05.
